# New Delhi metallo-β-lactamase-1-producing *Citrobacter portucalensis* belonging to the novel ST264 causing fatal sepsis in a vulnerable migratory sea turtle

**DOI:** 10.1016/j.onehlt.2023.100590

**Published:** 2023-06-22

**Authors:** Fábio P. Sellera, Danny Fuentes-Castillo, Bruna Fuga, Daphne W. Goldberg, Cristiane K.M. Kolesnikovas, Nilton Lincopan

**Affiliations:** a Department of Internal Medicine, School of Veterinary Medicine and Animal Science, University of São Paulo, São Paulo, Brazil; b School of Veterinary Medicine, Metropolitan University of Santos, Santos, Brazil; c One Health Brazilian Resistance Project (OneBR), São Paulo, Brazil; d Departamento de Patología y Medicina Preventiva, Facultad de Ciencias Veterinarias, Universidad de Concepción, Chillán, Chile; e Department of Clinical Analysis, Faculty of Pharmacy, University of São Paulo, São Paulo, Brazil; f Department of Microbiology, Instituto de Ciências Biomédicas, University of São Paulo, São Paulo, Brazil; g R3 Animal, Florianópolis, Santa Catarina, Brazil

**Keywords:** Antimicrobial resistance, Carbapenemase, *Lepidochelys olivacea*, NDM, Nosocomial bacteria, Wildlife

## Abstract

Olive ridley (*Lepidochelys olivacea*) turtles migrate across tropical regions of the Atlantic, Pacific, and Indian Oceans. Worryingly, olive ridley populations have been declining substantially and is now considered a threatened species. In this regard, habitat degradation, anthropogenic pollution, and infectious diseases have been the most notorious threats for this species. We isolated a metallo-β-lactamase (NDM-1)-producing *Citrobacter portucalensis* from the blood sample of an infected migratory olive ridley turtle found stranded sick in the coast of Brazil. Genomic analysis of *C. portucalensis* confirmed a novel sequence type (ST), named ST264, and a wide resistome to broad-spectrum antibiotics. The production of NDM-1 by the strain contributed to treatment failure and death of the animal. Phylogenomic relationship with environmental and human strains from African, European and Asian countries confirmed that critical priority clones of *C. portucalensis* are spreading beyond hospital settings, representing an emerging ecological threat to marine ecosystems.

## Highlights

• A fatal sepsis by a multidrug-resistant Gram-negative bacillus in a sea turtle was investigated.
• Identification of NDM-1 *Citrobacter portucalensis* belonging to the novel ST264 is highlighted.
• Clonal relatedness with *C. portucalensis* strains isolated from humans, animals, and the environment was examined.
• Global spread of NDM-producing bacteria in wildlife is presented.
• The ecological threat of carbapenemase-producing bacteria in the marine ecosystem is discussed.

## Graphical abstract

**Figure:**
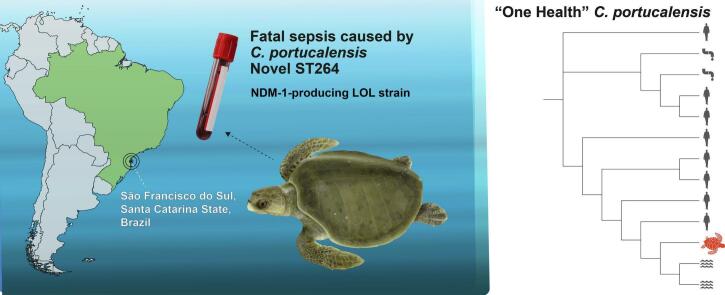

## 1 Introduction

The dissemination of carbapenemase-producing Enterobacterales beyond hospital settings has been a matter of ecological apprehensions [[1], [2], [3]], since most infections are associated with unfavorable clinical outcomes [4,5]. Antibiotic-resistant bacteria are found in people, animals, food and in the environment around the world. Although, some studies have reported the presence of carbapenemase-producing bacteria in coastal waters [[6], [7], [8], [9], [10], [11]], marine wildlife has been overlooked in the epidemiology of WHO critical priority pathogens. However, since anthropogenic pollution of aquatic ecosystems has been a growing global phenomenon, increasing rates of clinically significant antibiotic-resistant bacteria in the marine environment and its wildlife could be expected [6]. Moreover, rapid genomic and phenotypic methods are currently available to accelerate the identification of carbapenemase producers, strengthening epidemiological surveillance of antimicrobial resistance at the human-animal-environmental interface [6,12,13].

Sea turtles are considered key organisms for ocean health, since they have a direct impact on other species, contributing to the primary interactions in the evolution, structure and dynamics of marine ecosystems [14]. Unfortunately, sea turtles have been exposed to different anthropogenic pollutants, including biological ones, such as pathogenic bacteria resistant to clinically relevant antibiotics [15]. Therefore, in addition to being a potential ecosystem health bioindicator, sea turtles are considered sentinels for monitoring the dissemination of antibiotic resistance in marine environments [16].

Here, we report the identification of a metallo-β-lactamase (NDM-1)-producing *Citrobacter portucalensis* from the blood culture of an infected free-living olive ridley turtle (*Lepidochelys olivacea*), highlighting the negative implications of human nosocomial pathogens in marine wildlife. In fact, the production of NDM-1 by the strain contributed to treatment failure and death of the olive ridley turtle, considered a vulnerable species by the IUCN [17].

## 2 Materials and methods

An adult female olive ridley turtle (39.8 kg; curved carapace length of 72 cm, and curved carapace width of 71.5 cm) was found stranded alive in a beach of São Francisco do Sul (−26.2938373, −48.5378855), located in the Northern coast of Santa Catarina State, Brazil. Physical examination revealed signs of weakness, severe dehydration (PCV of 48%), buoyancy disorders, and difficulty in surfacing to breathe. The turtle was in good body condition; however it exhibited multiple abrasions and ulcers on the plastron and posterior flippers. Blood samples were collected from the dorsal cervical sinus for hematological examination and microbiological analysis.

Bacterial identification was determined by VITEK® 2 system (Biomerieux, USA), being further investigated by whole genome sequencing (WGS). Antimicrobial susceptibility testing was performed by VITEK® 2 system (Biomerieux, USA), disc diffusion, and/or Etest, methods [18], including amoxicillin/clavulanic acid, cefepime, ceftazidime, cefotaxime, ceftiour, gentamicin, ertapenem, imipenem, meropenem, trimethoprim/sulfamethoxazole, ciprofloxacin, and nalidixic acid. Metallo-β-lactamase production was investigated by imipenem-EDTA disk test [19]. *Escherichia coli* ATCC 25922 was used as control strain.

The animal was empirically treated with ceftazidime (20 mg/kg q. 72 h) administered intravenously. Unfortunately, despite continued medical therapy, the animal succumbed to death after initial supportive care. Necropsy revealed the loss of epidermis and bacterial colonies associated with moderate inflammatory infiltrate on the superficial dermis. Additionally, hydropic degeneration and necrosis were observed in both kidneys, which was probably associated with septic shock.

We performed WGS of the Gram-negative bacilli (LOL strain) using an Illumina NextSeq platform (Illumina, San Diego, CA, USA). The raw data was trimmed by TrimGalore v0.6.5 (https://github.com/FelixKrueger/TrimGalore) and assembled using Unicycler v0.4.8 (https://github.com/rrwick/Unicycler). We identified antimicrobial resistance genes, multilocus sequence typing (MLST) and plasmid incompatibility groups of *C. portucalensis* strain LOL using bioinformatic tools, available from the Center for Genomic Epidemiology (http://genomicepidemiology.org/). The Comprehensive Antibiotic Resistance Database – CARD – was also used to predict genes encoding efflux pumps (https://card.mcmaster.ca/home). NCBI Prokaryotic Genome Annotation Pipeline (PGAP) v4.10 (http://www.ncbi.nlm.nih.gov/genome/annotation_prok) was used for genome annotation.

## 3 Results and discussion

The Gram-negative bacilli (LOL strain) recovered from the blood sample was initially identified as *Citrobacter* spp. by VITEK® 2 system, being further confirmed as *C. portucalensis* by WGS. LOL strain displayed a multidrug-resistant profile [20] to amoxicillin/clavulanic acid, cefepime, ceftazidime, cefotaxime, ceftiour (MIC >32 μg/mL), gentamicin (MIC ≥64 μg/mL), ertapenem (MIC = 4 μg/mL), imipenem, meropenem, trimethoprim/sulfamethoxazole (MIC ≥320 μg/mL), ciprofloxacin (MIC ≥4 μg/mL), and nalidixic acid. Metallo-β-lactamase production was confirmed by imipenem-EDTA disk test.

Genomic data of *C. portucalensis* LOL strain (GenBank accession number: JAECZE000000000.1) confirmed the presence of *bla*_NDM-1_ gene, and revealed other resistant determinants conferring resistance to β-lactams (*bla*_CMY-13_, and *bla*_OXA-1_), aminoglycosides [*aph(3″)-Ib*, *aph(3′)-VI*, and *aph(6)-Id*], macrolides (*mphA*), rifampicin (*arr-3*), trimethoprim (*dfrA21*), tetracycline (*tetD*), quinolones [*aac(6′)-Ib-cr*], sulphonamide (*sul1* and *sul2*), and phenicols (*catB3*). The *bla*_NDM-1_ was flanked upstream by a *ble*_MBL_ gene (encoding a novel bleomycin resistance protein - BRP) and downstream by mobile element and *aph(3′)-VI* gene. CARD results showed several antibiotic efflux pump factors (*CRP, msbA, mdtB, mdtC, acrA, baeR, H-NS, emrB, emrR,* and *marA* genes) using over 90% of identity threshold. MLST analysis revealed that this strain belonged to a novel sequence type (ST), named ST264, and IncFIB(K)-like plasmid replicon was detected.

**Fig. 1:**
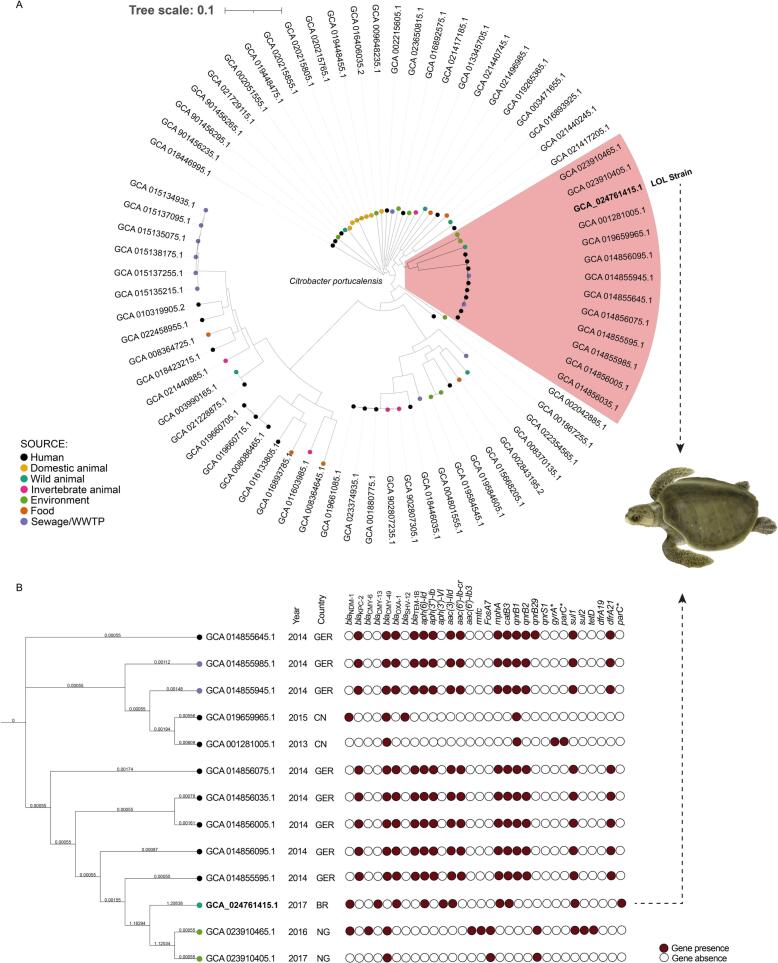
SNP-based phylogenomic analysis. A, phylogenomic tree of LOL strain and other *Citrobacter portucalensis* genome assemblies available at NCBI database with epidemiological information. B, phylogenomic tree of the highlighted cluster comparing individual antimicrobial resistance genes, and isolation source, year, and country. *, mutations in the quinolone resistance-determining region; GER, Germany; CN, China; BR, Brazil; NG, Nigeria.

We analyzed an additional 72*C. portucalensis* whole-genome sequences available from the Assembly database in the National Biotechnology Information Center (NCBI) that had epidemiological data (source, year, and country of collection). The phylogenomic relationship of these available *C. portucalensis* genome assemblies and LOL strain was evaluated based on single nucleotide polymorphism (SNP) using the CSI Phylogeny 1.4 from Center of Genomic Epidemiology (https://cge.food.dtu.dk/services/CSIPhylogeny/) (Fig. 1A). The figure was generated using the iTOL v.6 (https://itol.embl.de). The LOL strain recovered from turtle clustered with environmental strains from Nigeria, and human strains from Germany and China (Fig. 1). Among the 73 genomes analyzed, SNP counts varied between 0 and 40,127 (Supplementary Table S1).

**Fig. 2:**
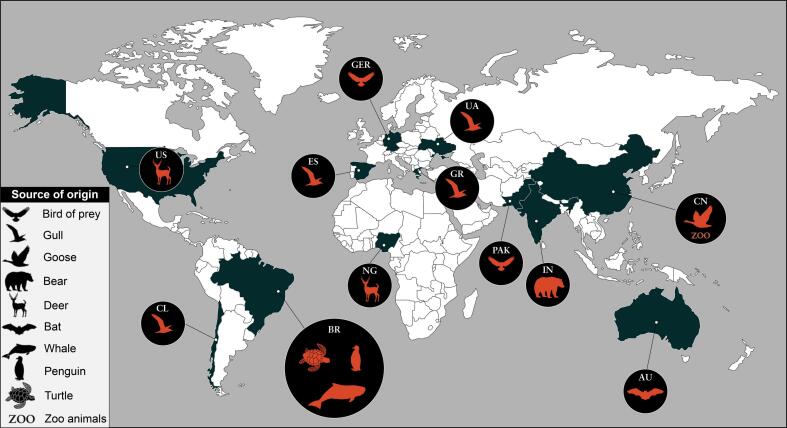
Epidemiological landscape of NDM-type-producing bacteria in wildlife. AU, Australia; BR, Brazil; CL, Chile; CN, China; NG, Nigeria; ES, Spain; GER, Germany; GR, Greece; IN, India; PAK, Pakistan; US, United States. Data were retrieved from PubMed database via the National Center for Biotechnology Information (NCBI) interface (accessed on August 19, 2022).

Antimicrobial resistance has been a global phenomenon that is no longer restricted to human healthcare settings. In this regard, wildlife may also play an important role in the epidemiology of antibiotic-resistant pathogens in the environment [[21], [22], [23]]. However, little is known about the clinical impact of these pathogens on wildlife and their ecological impacts on threatened species [1]. Of epidemiological concern, reports of the occurrence of NDM-producing bacteria in wildlife have begun to be globally reported. After a systematic search of the scientific literature documenting the occurrence of NDM-type-producing bacteria in wildlife, we observed that an increase in numbers of bacteria of this sort occurred in the last 5 years (Fig. 2). In this study, we report the further occurrence of NDM-1-producing *C. portucalensis* belonging to the novel ST264 causing fatal septicemia in a migratory sea turtle, which also constitute the first of report of NDM-type-positive *C. portucalensis* infection in wild animals, underlining that problems related to the dissemination of these bacteria in wildlife are much more complex than gut colonization.

Olive ridley turtles are the most abundant of all sea turtle species, being found throughout tropical and subtropical waters of the Atlantic, Indian and Pacific oceans [17]. According to the International Union for Conservation of Nature (IUCN), they are classified as vulnerable, mostly because like other sea turtle species, they are prone to population declines due to anthropogenic impacts. Although the active lifestyle of olive ridley turtles is predominantly pelagic, they could be easily found in coastal waters, including those affected by human activities [17]. There are many important nesting and feeding grounds on the east Pacific coast from as far north as Canada to as far south as southern Peru. In Brazil, it has been suggested that the offshore waters of the north-eastern Brazilian coast are a preferential habitat for olive ridley turtles, whereas this species might be using the north-eastern coast of Rio de Janeiro state (south east of Brazil) for feeding purposes or as part of its route to more southern foraging zones [24].

In this study, comparative sequence analysis included publicly available *C. portucalensis* genomes. To date, there are only two *C. portucalensis* strains (TV06 and ECTV13) producing ESBLs isolated from green sea turtles (*Chelonia mydas*), in Brazil [25], which are available in the NCBI database (Assembly accession numbers: GCA_008370135.1 and GCA_008370125.1); whereas no *C. portucalensis* strains have been identified in human hosts, in this country, so far. On the other hand, data from 4 environmental *C. portucalensis* strains (TK288, EK291, EC_47, and EC_49) isolated from water samples of beaches and the electronic waste dumpsite, in Nigeria, were also included (Assembly accession numbers: GCA_023910405.1, GCA_023910465.1, GCA_019584605.1, and GCA_019584545.1). In this regard, *C. portucalensis* from Brazil and Nigeria were clonally unrelated (21644–26691 SNPs differences) to the NDM-1-producing LOL strain. However, the migratory behavior of olive ridley turtles could be a contributing factor for acquiring multidrug-resistant bacteria in a country and then spreading in other regions.

Considering that *Citrobacter* spp. have been also recognized as important pathogens for wild sea turtles [26], the acquisition of carbapenemase genes could increase unfavorable clinical outcomes for turtles and other marine species. These findings suggest that dissemination of NDM-producing organisms may represent a new ecological threat to marine ecosystems with potentially serious clinical implications to wildlife inhabiting. Hence, we strongly encourage that continued surveillance of carbapenemase-producing bacteria in marine environments should be performed globally for a better comprehension of the transmission pathways and clinical impacts of such pathogens in marine populations.

The following is the supplementary data related to this article.

Supplementary Table S1 SNP matrix of analyzed *Citrobacter portucalensis* genome assemblies. Supplementary Table S1

## Ethics statement

The authors confirm that the ethical policies of the journal, as noted on the journal's author guidelines page, have been adhered to. No ethical approval was required for this specific study.

## Funding statement

This study was supported by the 10.13039/100000865Bill and Melinda Gates Foundation (Grand Challenges Explorations Brazil OPP1193112). Under the grant conditions of the Foundation, a CC BY or equivalent license is applied to the author accepted manuscript version arising from this submission. Additionally, this study was supported by the 10.13039/501100001807Fundação de Amparo à Pesquisa do Estado de São Paulo (2020/08224–9), 10.13039/501100003593Conselho Nacional de Desenvolvimento Científico e Tecnológico (AMR 443819/2018–1, 312249/2017–9, 422984/2021–3, and 314336/2021–4). NL is a research fellow of 10.13039/501100003593CNPq (314336/2021–4). BF was a research fellow of PNPD/CAPES (88887.358057/2019–00).

## CRediT authorship contribution statement

**Fábio P. Sellera:** Conceptualization, Methodology, Data curation, Investigation, Writing – original draft, Writing – review & editing. **Danny Fuentes-Castillo:** Conceptualization, Methodology, Data curation, Writing – original draft, Writing – review & editing. **Bruna Fuga:** Conceptualization, Methodology, Data curation, Investigation, Writing – original draft, Writing – review & editing. **Daphne W. Goldberg:** Conceptualization, Methodology, Data curation, Investigation, Writing – original draft, Writing – review & editing. **Cristiane K.M. Kolesnikovas:** Methodology, Investigation, Writing – review & editing. **Nilton Lincopan:** Conceptualization, Resources, Writing – review & editing, Funding acquisition.

## Declaration of Competing Interest

All authors declare no conflicts of interest.

## Data Availability

The data that support the findings of this study are available from the corresponding author upon reasonable request. This Whole Genome Shotgun project has been deposited at DDBJ/ENA/GenBank under the accession JAECZE000000000.1.
